# The Combined Heterozygosity of Factor V Leiden and G20210A Prothrombin Gene Mutation in a Patient With Venous Thromboembolism

**DOI:** 10.7759/cureus.44835

**Published:** 2023-09-07

**Authors:** Marcia Machado, Marta Cunha, Filipe Gonçalves, Carlos Fernandes, Jorge Cotter

**Affiliations:** 1 Internal Medicine, Hospital da Senhora da Oliveira, Guimarães, PRT

**Keywords:** prothrombin g20210a factor ii mutation, heterozygous factor v leiden mutation, deep vein thrombosis (dvt), acute pulmonary embolism, venous thromboembolism (vte)

## Abstract

Venous thromboembolism (VTE) is a chronic illness that includes pulmonary embolism (PE) and deep vein thrombosis (DVT), and many risk factors are associated. Anticoagulation therapy remains the cornerstone of venous thromboembolism management, and the duration of anticoagulation depends on the risk of venous thromboembolism.

We report a case of a female with a combined heterozygosity of factor V Leiden and G20210A prothrombin gene mutation.

## Introduction

Venous thromboembolism (VTE) is a potentially fatal medical condition that includes pulmonary embolism (PE) and deep vein thrombosis (DVT) and has an incidence of approximately 10 million people yearly. Our understanding of VTE has dramatically improved in recent years due to advancements in diagnostic technologies and extensive research. The risk factors include prolonged immobility, malignancy, major surgery, estrogen therapy, and inherited and acquired thrombophilia [1]. The inherited disorders include factor V Leiden, G20210A prothrombin mutation, deficiencies in natural anticoagulants (protein C, protein S, and antithrombin), elevated clotting factors such as factors VIII and XI, and hyperhomocysteinemia [2]. About 5% of the population has factor V Leiden. Heterozygotes have a threefold higher risk of VTE than the general population, while homozygotes face a significantly elevated risk, ranging from 50 to 80 times higher than the general population. On the other hand, heterozygous prothrombin mutation is present in about 2% [3] of the population and increases the risk of venous thrombosis by 2.5 times. The choice of anticoagulation can be challenging due to the need for studies among different types of thrombophilias [4].

We present the case of a 31-year-old female with pulmonary venous thromboembolism and venous deep vein thrombosis with a combined heterozygosity of factor V Leiden and G20210A prothrombin gene mutation who was prescribed enoxaparin acutely and direct oral anticoagulants (DOACs) indefinitely.

## Case presentation

A 31-year-old female patient presented to the emergency department with significant pain in her left lower limb, persisting for two days. She also reported sudden-onset pleuritic chest pain intensity of 8/10 with no relief after analgesics and dyspnea, which had developed for one day. She had no other symptoms. The patient had no previous comorbidities, and her family history did not reveal any noteworthy medical conditions. She smoked one pack daily for five years and took combined estrogen-progestin oral contraceptives. She denied taking other medications or supplements.

**Figure 1:**
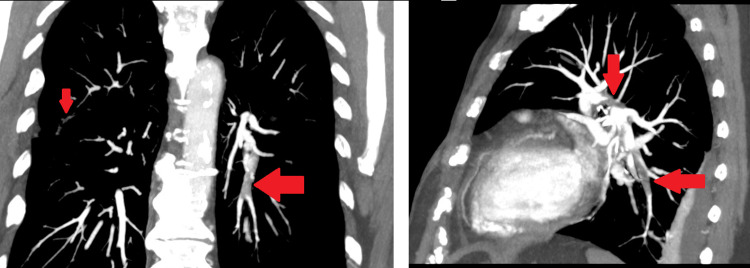
Coronal and sagittal chest CT showing bilateral pulmonary embolism (arrows)

**Figure 2:**
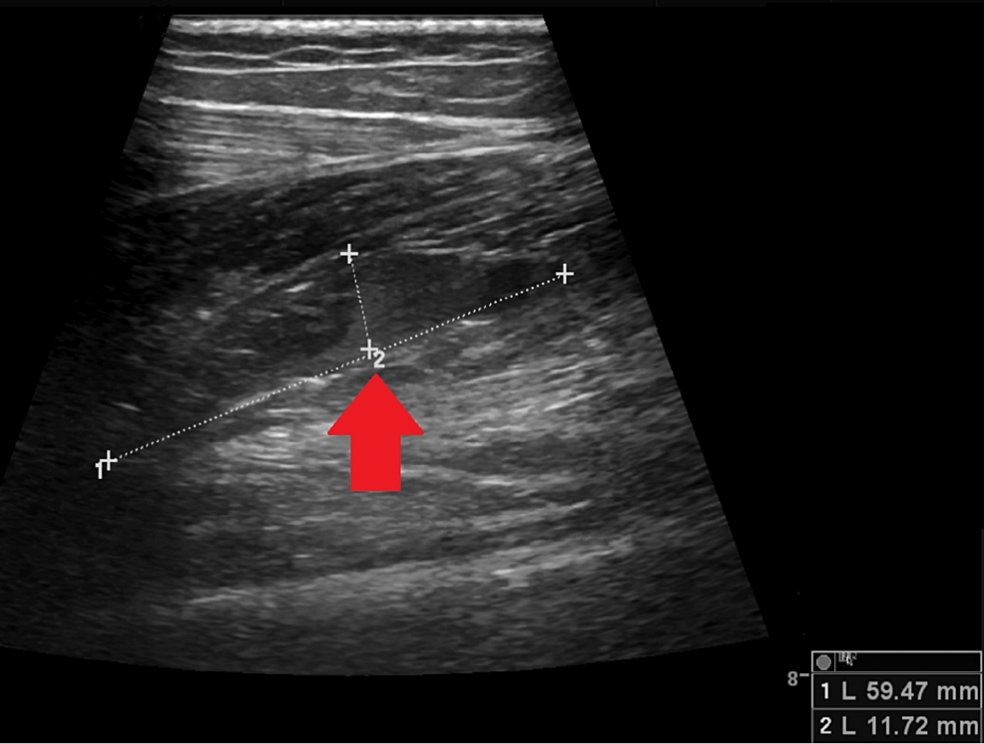
Lower limb Doppler imaging Acute venous thromboembolism (arrow) in the left popliteal vein

On admission, the patient was diaphoretic and dyspneic; her blood pressure was 110/76 mmHg, her heart rate was 110 beats per minute, her oxygen saturation level in room air was 97%, and her respiratory rate was 26 breaths per minute. During the physical examination, the only notable difference was that the left lower limb presented signs of deep vein thrombosis. The blood test showed no significant findings except for a D-dimer level of 563 ng/mL (the reference concentration of D-dimer is below 250 ng/mL). A Doppler scan revealed an acute venous thromboembolism in the left popliteal vein, and the CT pulmonary angiography revealed a bilateral pulmonary embolism affecting the segmental branches (Figures 1, 2).

She was admitted and anticoagulated with 1 mg/kg of enoxaparin every 12 hours. She was tested for factor V Leiden and prothrombin gene mutation, and this revealed a heterozygosity of factor V Leiden and prothrombin gene mutation. Occult malignancy had been ruled out. After being discharged, the patient received a prescription for a vitamin K antagonist (VKA) medication.

Months later, she completed a thrombophilia study that revealed no changes except the previously identified mutation and switched from warfarin to DOAC. Up to the present, there have been no recent thrombotic events.

## Discussion

Venous thromboembolism has variable morbidity and mortality. After the initial diagnosis of VTE, the recurrence rate within 10 years is estimated at around 25%, with a peak occurring at six months and a gradual decline afterward. However, this rate can vary depending on the thrombosis's etiology [5].

The pathophysiology of VTE can be attributed to three main factors known as Virchow's triad: venous stasis, vessel wall injury, and hypercoagulability, which are known to increase the risk of VTE development [6]. There are several contributing factors to venous thromboembolism. The strong risk factors include major surgery, prolonged immobilization, and active cancer. However, weak factors (e.g., chronic inflammatory disorders, estrogen therapy, or pregnancy) or no apparent cause can also lead to the disease. The precise role of genetics in the risk of venous thromboembolism has yet to be fully elucidated [1].

Symptoms may vary depending on the affected vein [6]. Patients with pulmonary embolism typically exhibit symptoms such as dyspnea (80% of cases), pleuritic chest pain (60%-70%), hemoptysis (5%-13%), or hypoxemia (70%). However, in some instances, they may also manifest severe hemodynamic compromise (10%-20%).

Performing a clinical assessment of patients to evaluate risk factors and identify signs and symptoms at the initial presentation is essential for estimating the likelihood of venous thromboembolism before conducting any additional investigations (the Wells score, D-dimer testing, and duplex ultrasound).

The objective of treating venous thromboembolism is to prevent the extension of the thrombus and the occurrence of embolization. Anticoagulation is the first-line treatment for most patients [1]. In some instances, thrombolysis can be a viable option for younger patients with submassive PE and a low risk of bleeding or those at high risk for decompensation due to other cardiopulmonary conditions. It is essential to closely monitor patients with submassive PE for any signs of hemodynamic compromise [7].

Laboratory workup for thrombophilia is not recommended routinely for determining the duration of therapy. The workup should be considered for patients under 50, particularly those with a robust family history of clotting disorders, thromboses in atypical locations, or recurrent episodes. The American Society of Hematology (ASH) guideline recommends thrombophilia testing to define the duration of anticoagulant treatment in females with no other risk factors for venous thromboembolism apart from using combined oral contraceptives. If thrombophilia is identified in these cases, it advises considering indefinite anticoagulant treatment [8].

The ASH guideline panel recommends using direct oral anticoagulants (DOACs) over vitamin K antagonists (VKAs) in most patients [7]. According to the literature, the data suggests that DOACs are safe and effective in treating patients with thrombophilia. However, the risk of thrombosis differs among subgroups of patients depending on genetic mutation, and more studies are needed [4].

## Conclusions

Genetic testing has revolutionized our understanding of hereditary thrombophilias, providing personalized management plans and risk assessments. This significant advancement emphasizes the importance of continued research and comprehensive prevention measures, especially in high-risk settings, to minimize the negative effects on individuals and healthcare systems. More research is required to determine the safety and effectiveness of treatments in patients with thrombophilia with different risks of thrombosis recurrence.
